# Long-term outcomes of a phase II randomized controlled trial comparing intensity-modulated radiotherapy with or without weekly cisplatin for the treatment of locally recurrent nasopharyngeal carcinoma

**DOI:** 10.1186/s40880-016-0081-7

**Published:** 2016-02-15

**Authors:** Ying Guan, Shuai Liu, Han-Yu Wang, Ying Guo, Wei-Wei Xiao, Chun-Yan Chen, Chong Zhao, Tai-Xiang Lu, Fei Han

**Affiliations:** Department of Radiation Oncology, Affiliated Cancer Hospital of Guangxi Medical University, Cancer Institute of Guangxi Zhuang Autonomous Region, Nanning, 530021 Guangxi P. R. China; Department of Radiotherapy Oncology, The Sixth Affiliated Hospital of Sun Yat-sen University, Guangzhou, 510655 Guangdong P. R. China; Department of Radiotherapy Oncology, Sun Yat-sen University Cancer Center, State Key Laboratory of Oncology in South China, Collaborative Innovation Center for Cancer Medical, 651 Dongfeng Road East, Guangzhou, 510060 Guangdong P. R. China; Department of Clinical Trial, Sun Yat-sen University Cancer Center, State Key Laboratory of Oncology in South China, Collaborative Innovation Center for Cancer Medical, Guangzhou, 510060 Guangdong P. R. China

**Keywords:** Recurrence, Nasopharyngeal carcinoma, Intensity-modulated radiation therapy, Concomitant chemoradiotherapy, Cisplatin

## Abstract

**Background:**

Salvage treatment for locally recurrent nasopharyngeal carcinoma (NPC) is complicated and relatively limited. Radiotherapy, combined with effective concomitant chemotherapy, may improve clinical treatment outcomes. We conducted a phase II randomized controlled trial to evaluate the efficacy of intensity-modulated radiotherapy with concomitant weekly cisplatin on locally recurrent NPC.

**Methods:**

Between April 2002 and January 2008, 69 patients diagnosed with non-metastatic locally recurrent NPC were randomly assigned to either concomitant chemoradiotherapy group (*n* = 34) or radiotherapy alone group (*n* = 35). All patients received intensity-modulated radiotherapy. The radiotherapy dose for both groups was 60 Gy in 27 fractions for 37 days (range 23–53 days). The concomitant chemotherapy schedule was cisplatin 30 mg/m^2^ by intravenous infusion weekly during radiotherapy.

**Results:**

The median follow-up period of all patients was 35 months (range 2–112 months). Between concomitant chemoradiotherapy and radiotherapy groups, there was only significant difference in the 3-year and 5-year overall survival (OS) rates (68.7% vs. 42.2%, *P* = 0.016 and 41.8% vs. 27.5%, *P* = 0.049, respectively). Subgroup analysis showed that concomitant chemoradiotherapy significantly improved the 5-year OS rate especially for patients in stage rT3–4 (33.0% vs. 13.2%, *P* = 0.009), stages III–IV (34.3% vs. 13.2%, *P* = 0.006), recurrence interval >30 months (49.0% vs. 20.6%, *P* = 0.017), and tumor volume >26 cm^3^ (37.6% vs. 0%, *P* = 0.006).

**Conclusion:**

Compared with radiotherapy alone, concomitant chemoradiotherapy can improve OS of the patients with locally recurrent NPC, especially those with advanced T category (rT3–4) and stage (III–IV) diseases, recurrence intervals >30 months, and tumor volume >26 cm^3^.

## Background

Local tumor control rates for primary nasopharyngeal carcinoma (NPC) have reached up to 80% with the advent of precise radiotherapy technologies including intensity-modulated radiotherapy (IMRT) and the use of combined chemoradiotherapy. Unfortunately, approximately 10% of patients with NPC develop local and regional recurrences after IMRT [1–3].

Salvage treatment for locally recurrent NPC is difficult, and treatment options, which include surgery, radiotherapy, and chemotherapy, are very limited. Surgery, stereotactic radiotherapy, and brachytherapy are usually limited by the extent of recurrent lesions and are used only for selected patients. Chemotherapy alone is generally recommended as a palliative treatment for advanced cases. A repeat course of external radiotherapy is generally prescribed for locally recurrent NPC. IMRT is a preferred treatment method because of its unique dosimetric properties. For the treatment of locally recurrent NPC with conventional radiotherapy, a dose of at least 60 Gy is recommended, as this dose is associated with improved control and/or survival during re-irradiation [4, 5]. Total dose of re-irradiation is restricted by many factors, and it is difficult to administer the same radiation dose as the initial course due to severe late complications [6, 7]. To address these issues, comprehensive treatment modalities for locally recurrent NPC, coupled with the minimum effective re-irradiation dose, should be considered in order to yield positive treatment outcomes and reduce radiation toxicity. NPC is a chemotherapy-sensitive disease. To date, a single or combined chemotherapy regimen of cisplatin and 5-fluorouracil has been established as the standard of care for NPC at the advanced and recurrent/metastatic stage. For newly diagnosed and metastatic NPC patients, a treatment regimen that includes cisplatin can result in a superior tumor response rate compared with regimens lacking cisplatin [8]. Some retrospective studies of concomitant chemoradiotherapy using cisplatin have been conducted for locally recurrent NPC [9, 10]. The results of these studies suggest that this form of combined modality treatment might be useful in salvaging patients with recurrent disease.

Therefore, we conducted a prospective, phase II randomized controlled trial to evaluate the efficacy of IMRT with concomitant weekly cisplatin on locally recurrent NPC.

## Patients and methods

### Patient eligibility

All participants were required to provide written informed consent before being enrolled in the study. The study protocol was approved by Sun Yat-sen University Cancer Center Review Board. Patients were eligible for the study if they met the following inclusion criteria: (a) superficial lesions histologically confirmed by biopsy, or deep-seated lesions such as those on the skull base or cavernous sinus supported by imaging studies, and clinical symptoms; (b) no evidence of distant metastases at diagnosis; (c) an interval of more than 6 months between the completion of primary radiotherapy and recurrence; (d) Karnofsky performance status score of at least 70; (e) adequate heart, lung, renal, and hepatic functions and absence of serious diseases; (f) white blood cell count ≥4.0 × 10^9^/L, neutrophil count ≥2.0 × 10^9^/L, platelet count ≥100 × 10^9^/L, hemoglobin ≥100 g/L, all liver function indicators ≤2.5 times the upper limit of normal, and creatinine clearance ≥60 mL/min; and (g) at least 18 years of age.

Patients were excluded if they met one of the following exclusion criteria: (a) prior chemotherapy or anti-epidermal growth factor receptor therapy, or prior surgery for recurrent NPC lesions; (b) contraindications for chemotherapy and radiotherapy, such as (1) severe cerebral, cardiac, or peripheral vascular disease, and severe chronic heart disease; (2) active or recent gastrointestinal bleeding; (3) diabetes mellitus with severe organ damage; (4) severe end organ damage; and (5) allergic to cisplatin; (c) no nasopharyngeal magnetic resonance imaging (MRI) within 3 months following the completion of radiotherapy or inability to undergo MRI scan; (d) women who were pregnant or lactating; and (e) patients with a second malignancy, with the exception of cured skin basal cell carcinoma or early-stage cervical cancer.

The cases were staged based on the detailed disease history, comprehensive clinical examination, routine blood count, blood biochemical profile including renal and hepatic functions, the detection of Epstein-Barr virus, electrocardiogram, and MRI of the nasopharynx and neck. Chest X-rays, abdominal ultrasonographic examinations, and whole-body isotope bone scans were also performed to detect distant metastases. Systemic 2-[F-18] fluoro-2-deoxy-d-glucose positron emission tomography/computed tomography (^18^F-FDG PET/CT) scans were performed for some patients. The disease at recurrence was staged according to the American Joint Commission on Cancer TNM staging system (6th edition, 2002) and was reported as rTx, rNx, and rTxNx in this paper.

### Random assignment

The registration and randomization procedures were carried out using a digital sheet. The randomization code was developed using a computerized random number generator. All patients were randomized with equal probability to concomitant chemoradiotherapy group or radiotherapy alone group.

### IMRT

The gross tumor volume (GTV) and cervical lymph node tumor volume (GTVnd) were defined as the gross extent of the tumor shown by CT/MRI and physical examinations. GTV and GTVnd were contoured according to the International Commission on Radiation Units and Measurements Report 62 (ICRU62) guidelines. The clinical target volume (CTV) was delineated, which included the GTV with a 1–1.5 cm margin. The CTV also included the entire nasopharyngeal space and the positive lymph node regions. When GTV was adjacent to critical organs at risk (OARs), such as the spinal cord or brainstem, the margin of CTV was adjusted to no more than 3 mm depending on the proximity of critical structures.

The prescribed irradiation dose for both groups was 60 Gy in 27 fractions, 2.2 Gy per fraction, with 5 daily fractions per week for approximately 5.5 weeks. A detailed description of OAR contouring and the procedure for delivering IMRT were provided in our previous reports [11, 12].

### Concomitant chemotherapy

The concomitant chemotherapy schedule was cisplatin 30 mg/m^2^ by intravenous infusion weekly, repeated on days 1, 8, 15, 22, 29, and 36 during radiotherapy. All patients received an antiemetic prophylaxis of 5-hydroxytryptamine-3 receptor antagonist. The dose of cisplatin was adjusted according to toxicity. In instances of grade 3 or 4 toxicity following chemotherapy, the dose of cisplatin was decreased by 25% at the subsequent drug administration. Chemotherapy was postponed or discontinued for patients who experienced serious toxicity and were unable to recover prior to the next scheduled drug administration.

### Assessment and follow-up

Patients were evaluated weekly during the treatment with detailed clinical examinations and observation of acute toxicities. Tumor response was evaluated by nasopharyngoscopy at the end of treatment. The following assessments were performed at the first 3-month visit: (1) physical examination, (2) MRI of the nasopharynx and neck, and (3) chest X-ray and ultrasound scan of the abdomen. Patients were followed every 3 months during the first 3 years and every 6 months thereafter. Tumor response was classified according to the Response Evaluation Criteria in Solid Tumors version 1.0. Radiotherapy toxicity was graded according to the Radiation Therapy Oncology Group scoring schema, taking toxicity after primary radiotherapy as a baseline assessment.

### Study endpoints

The primary endpoint of this study was overall survival (OS), which was determined from the date of treatment initiation to the date of death for any cause or the date of last follow-up.

The secondary endpoints included locoregional failure-free survival (LFFS), distant metastasis-free survival (DFFS), and treatment toxicity. LFFS was determined from the date of treatment initiation to the date of detection of locoregional recurrence or the date of last follow-up. DFFS was determined from the date of treatment initiation to the date of detection of distant metastases or the date of last follow-up.

### Statistical analysis

Based on previous studies, the reported 5-year OS rate of patients with locally recurrent NPC was approximately 20% [13, 14]. We hypothesized that the use of IMRT combined with cisplatin weekly could improve the 5-year OS rate by 30%. The sample size was calculated based on the mean difference-based test, with α = 0.05, 1 − β = 0.8, and 1:1 into groups. This power level, with an expected loss of 5% of patients during follow-up, indicated that the study required a sample size of at least 70 patients (35 per group). Data analyses were carried out using SPSS 21.0 statistical software. The OS, LFFS, and DFFS curves were computed by the Kaplan–Meier method and compared by the log-rank test. The χ^2^ test (or Fisher’s exact test, if indicated) was used to compare the adverse events and other categorical variables between the two treatment groups. We performed multivariate analysis with the Cox proportional hazards model to test the independent significance of different factors. All tests were two-sided, and *P* values of less than 0.05 were considered significant.

## Results

### Patients

**Fig. 1:**
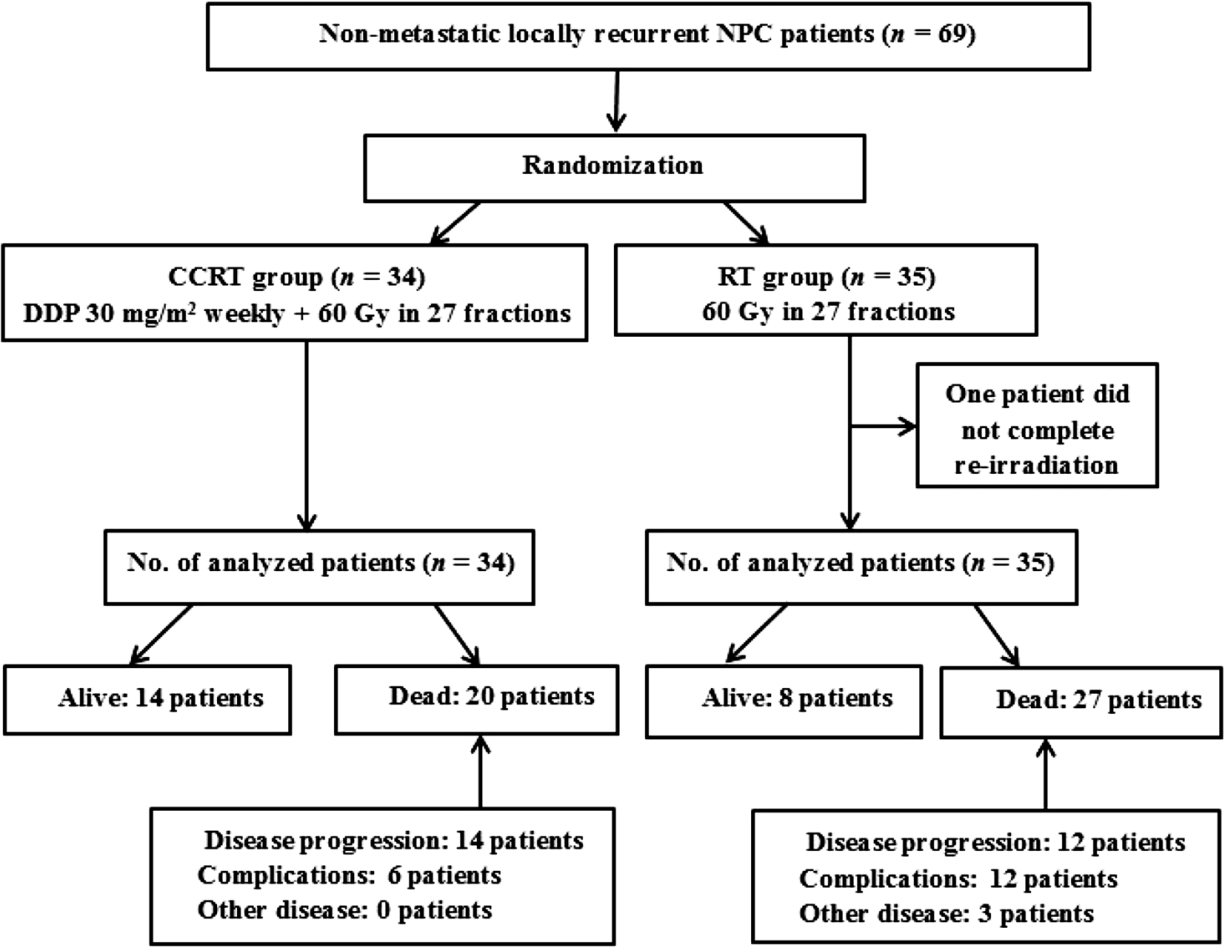
Flowchart for trial participants of the study. *NPC* nasopharyngeal carcinoma, *CCRT* concomitant radiochemotherapy, *RT* radiotherapy alone, *DDP* cisplatin, *No.* number

Between April 2002 and January 2008, 69 patients were enrolled in the study and randomly assigned to the concomitant chemoradiotherapy group (*n* = 34) or the radiotherapy alone group (*n* = 35). The two treatment groups were well balanced in terms of baseline demographics and clinical characteristics (Table 1). The flowchart of the study design is shown in Fig. 1.

**Table 1:** Baseline characteristics of 69 patients with non-metastatic locally recurrent nasopharyngeal carcinoma (NPC)

Characteristic	CCRT group	RT group	*P* value^a^
	(*n* = 34)	(*n* = 35)	
Sex (cases)			0.490
Male	27	30	
Female	7	5	
Age (years)			0.812
Range	31–63	32–73	
Median	42	48	
Interval of recurrence (months)			0.587
Range	12–121	9–72	
Median	26.5	30.0	
Initial radiation dose (Gy)			0.545
Range	66–78	68–86	
Median	70	72	
Pathology (cases)			0.532
WHO type II	2	3	
WHO type III	27	24	
Diagnosed by clinical diagnosis (cases)	5	8	
rT category^b^			0.175
rT1	2	3	
rT2	3	7	
rT3	17	10	
rT4	12	15	
rN category^b^			0.778
rN0	30	29	
rN1	3	5	
rN2	1	1	
Clinical stage^b^ (cases)			0.448
I	2	3	
II	4	7	
III	16	10	
IV	12	15	
Pre-treatment serious complications^c^ (cases)			0.618
No	26	27	
Yes	8	8	
Tumor volume (cm^3^)			0.242
Range	3–146	7–116	
Median	28	29	
Initial treatment modality (cases)			0.543
Radiochemotherapy	11	9	
Radiotherapy alone	23	26	
Initial irradiation technique (cases)			0.218
2D	34	32	
3D-CRT	0	2	
IMRT	0	1	

*WHO* World Health Organization, *2D* two-dimension conventional radiotherapy, *3D-CRT* three-dimension conformal radiotherapy, *IMRT* intensity-modulated radiotherapy, *CCRT* concomitant chemoradiotherapy, *RT* radiotherapy alone

^a^By χ^2^ test

^b^Restage according to the 2002 6th edition TNM staging system of the American Joint Commission on Cancer (AJCC)

^c^Pre-treatment serious complications include trismus, radiation encephalopathy, and posterior cranial nerve palsies

### Acute toxicity

No grade 5 toxicity (death) occurred during treatment. All patients in the concomitant chemoradiotherapy group completed the scheduled radiotherapy course. A single patient in the radiotherapy alone group did not complete the scheduled irradiation for personal reason (total radiotherapy dose 39.6 Gy in 18 fractions for 23 days). This patient was still included in the statistical analysis according to the intention-to-treat analysis principle. Of the 34 patients in the concomitant chemoradiotherapy group, 2.9% (*n* = 1), 14.7% (*n* = 5), 76.5% (*n* = 26), and 5.9% (*n* = 2) of patients completed 4, 5, 6, and 7 cycles of cisplatin, respectively. In result, all 34 patients completed total 199 cycles of concomitant chemotherapy during radiotherapy. Eight of the 34 patients (23.5%) in the concomitant chemoradiotherapy group and six of the 35 patients (17.1%) in the radiotherapy alone group experienced grades 3–4 toxic effects (χ^2^ = 1.252, *P* = 0.263). In the concomitant chemoradiotherapy group, four patients experienced grades 3 and 4 hematologic toxicities: leukopenia (*n* = 3) and neutropenia (*n* = 1). No patients in the radiotherapy alone group experienced grades 3 and 4 leukopenia or neutropenia.

### Follow-up

The period of follow-up was calculated from the initial day of treatments. By July 2013, the median follow-up was 35 months (range 2–112 months).

### Survival outcomes

**Fig. 2:**
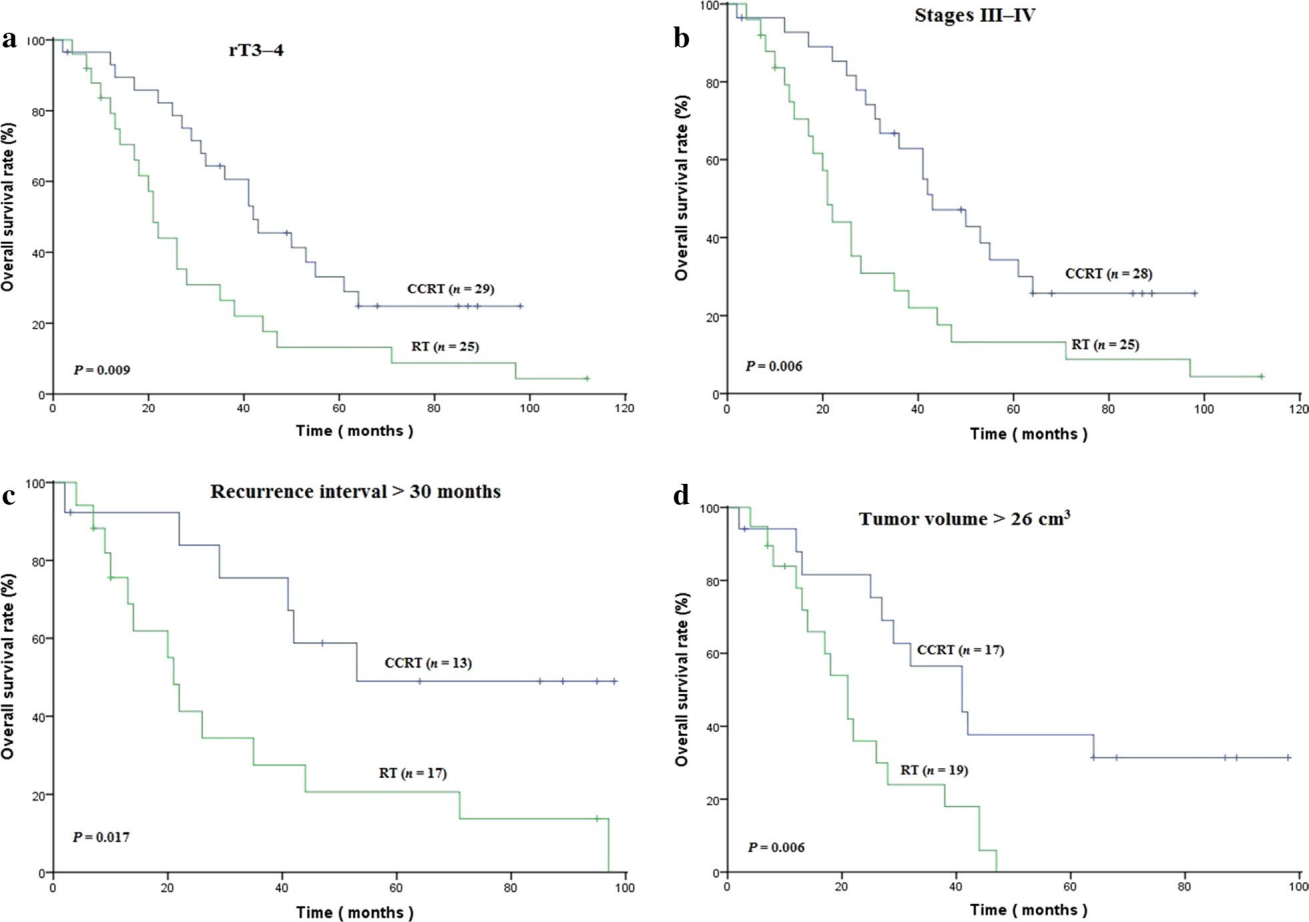
Kaplan–Meier curves of overall survival in distinct subgroups of NPC patients treated with CCRT or RT. The results show that CCRT significantly improved the 5-year overall survival rate for patients with **a** rT3–4 category disease (33.0% vs. 13.2%, *P* = 0.009), **b** stages III–IV NPC (34.3% vs. 13.2%, *P* = 0.006), **c** recurrence interval >30 months (49.0% vs. 20.6%, *P* = 0.017), and **d** tumor volume >26 cm^3^ (37.6% vs. 0%, *P* = 0.006) as compared with RT

A comparison of the two treatment groups (the concomitant chemoradiotherapy group vs. the radiotherapy alone group) revealed no significant differences in 3-year or 5-year LFFS rate (56.2% vs. 62.4%, *P* = 0.848 and 43.4% vs. 62.4%, *P* = 0.502, respectively) and DFFS rate (90.3% vs. 81.4%, *P* = 0.0.341 and 85.8% vs. 81.4%, *P* = 0.519, respectively). Concomitant chemoradiotherapy significantly improved 3-year and 5-year OS rates compared with radiotherapy alone (68.7% vs. 42.4%, *P* = 0.016 and 41.8% vs. 27.5%, *P* = 0.049, respectively). Subgroup analysis showed that concomitant chemoradiotherapy significantly improved the 5-year OS rate especially for patients with category rT3–4 disease (33.0% vs. 13.2%, *P* = 0.009), stages III–IV NPC (34.3% vs. 13.2%, *P* = 0.006), recurrence interval >30 months (49.0% vs. 20.6%, *P* = 0.017), and tumor volume >26 cm^3^ (37.6% vs. 0%, *P* = 0.006), as shown in Fig. 2.

### Multivariate analyses

Multivariate analyses of prognostic factors were performed using a Cox regression model. Univariate and multivariate analysis results are shown in Tables 2 and 3. In the multivariate analysis, only age, rT category, clinical stage, and treatment were independent prognostic factors for OS.

**Table 2:** Univariate analysis of overall survival (OS) for potential prognostic factors of patients with non-metastatic locally recurrent NPC

Variable	Entire cohort (69 patients)		χ^2^	*P* value^a^	CCRT group (34 patients)		RT group (35 patients)		χ^2^	*P* value^a^
	*n*	5-year OS rate (%)			*n*	5-year OS rate (%)	*n*	5-year OS rate (%)		
Sex			0.810	0.368						
Male	57	33.4			27	37.0	30	28.9	1.941	0.164
Female	12	41.7			7	57.1	5	20.0	2.849	0.091
Age (years)			9.656	0.002						
≤46	38	46.3			22	43.0	16	50.0	0.039	0.843
>46	31	19.9			12	38.5	19	10.5	3.385	0.066
rT category^b^			10.342	0.001						
rT1–2	15	72.2			5	100	10	60.0	2.725	0.099
rT3–4	54	24.2			29	33.0	25	13.2	6.789	0.009
rN category^b^			0.049	0.824						
rN0	59	33.2			30	40.1	29	25.2	3.614	0.057
rN1–2	10	44.4			4	50.0	6	40.0	0.161	0.688
Clinical stage^b^			8.613	0.003						
I–II	16	67.7			6	83.3	10	60.0	1.042	0.307
III–IV	53	24.7			28	34.3	25	13.2	7.574	0.006
Recurrence interval (months)			0.343	0.558						
≤30	39	36.0			21	37.5	18	33.3	0.126	0.723
>30	30	33.4			13	49.0	17	20.6	5.664	0.017
Tumor volume (cm^3^)			9.352	0.002						
≤26	33	51.7			17	42.9	16	56.3	0.023	0.881
>26	36	18.3			17	37.6	19	0.0	7.482	0.006

Abbreviations as in Table 1

^a^By log-rank test

^b^Restage according to the 2002 6th edition TNM staging system of the American Joint Commission on Cancer (AJCC)

**Table 3:** Multivariate Cox regression model of overall survival of patients with non-metastatic locally recurrent NPC

Variable	*β*	Relative risk	*P* value^a^	Hazard ratio	95% confidence interval
rT1–2 category vs. rT3–4 category^b^	4.592	1.219	0.000	98.668	9.053–1075.343
Stages I–II versus Stages III–IV^b^	–2.802	1.098	0.011	0.061	0.007–0.522
CCRT versus RT	0.942	0.313	0.003	2.566	1.389–4.740
≤46 years versus >46 years	0.990	0.309	0.001	2.692	1.470–4.931

Abbreviations as in Tables 1 and 2

^a^By Cox proportional hazards model test

^b^Restage according to the 2002 6th edition TNM staging system of AJCC

### Late toxicity and causes of death

No differences were noted in incidences of late toxicities such as nasopharyngeal mucosal necrosis, radiation encephalopathy, and cranial nerve palsies between the two groups. The incidences of nasopharyngeal hemorrhage and trismus were significantly lower in the concomitant chemoradiotherapy group than in the radiotherapy alone group (8.8% vs. 34.3%, *P* = 0.010; 0% vs. 14.3%, *P* = 0.022), as shown in Table 4.

**Table 4:** Morbidity events and causes for death

Item	CCRT group (34 patients)	RT group (35 patients)	χ^2^	*P* value^a^
	*n* (%)	*n* (%)		
*Morbidity and events*				
Nasopharyngeal massive hemorrhage	3 (8.8)	12 (34.3)	6.572	0.010
Nasopharyngeal mucosal necrosis	8 (23.5)	13 (37.1)	1.510	0.219
Radiation encephalopathy	4 (11.8)	5 (14.3)	0.097	0.756
Trismus	0 (0.0)	5 (14.3)	5.237	0.022
Anterior cranial nerve palsies	1 (2.9)	2 (5.7)	0.319	0.572
Posterior cranial nerve palsies	1 (2.9)	2 (5.7)	0.319	0.572
*Causes for death*				
Recurrence	10 (29.4)	7 (20.0)	0.823	0.364
Distant metastasis	1 (2.9)	3 (8.6)	1.001	0.317
Rrecurrence + distant metastasis	2 (5.9)	2 (5.7)	0.001	0.976
Residual disease + progression	1 (2.9)	0 (0.0)	1.045	0.307
Other diseases	0 (0.0)	3 (8.6)	3.047	0.081
Nasopharyngeal hemorrhage	2 (5.9)	10 (28.6)	6.180	0.013
Radiation encephalopathy	4 (11.8)	2 (5.7)	0.795	0.373

^a^By χ^2^ test

## Discussion

In our study, concomitant chemoradiotherapy significantly improved 3-year and 5-year OS rates compared with radiotherapy alone. Subgroup analysis showed that concomitant chemoradiotherapy significantly improved the 5-year OS rate especially for patients in the category rT3–4, stages III–IV, recurrence interval >30 months, and tumor volume >26 cm^3^.

Concomitant chemoradiotherapy can enhance tumor control, act as a radiotherapy sensitizer, and eradicate distant micrometastases. Most locally recurrent NPCs have a similar pathology to primary tumors, with local recurrence occurring predominantly at the advanced T category [15]. Tumor in locally recurrent NPC is often poorly sensitive to radiotherapy, which can be attributed to tissue fibrosis and vascular changes. Therefore, radiotherapy, combined with effective concomitant chemotherapy, may improve clinical treatment outcomes.

Tumor remission rates following treatment with either a single agent or combination chemotherapy have been shown to be 10%–30% and 40%–50%, respectively; however, OS was only extended 5–6 months [16]. Previous studies demonstrated that re-irradiation of locally recurrent NPC resulted in poor outcomes, with reported 5-year OS rates ranging from 5.8% to 40.0% and local control rates ranging from 14 to 61% [4, 13, 17]. The advent of IMRT has offered the potential of improving dose and conformation of radiation to the target, thus sparing critical structures. The clinical advantages of IMRT as a salvage treatment with respect to both disease control and adverse effect profiles have been demonstrated for locally recurrent NPC. Although IMRT can achieve control in 71.0% –85.8% of cases, it is associated with a high incidence of late complications that are attributable to excessively high doses, which occur in 26% –80% of cases; these complications include nasopharyngeal mucosal necrosis/massive hemorrhage and radiation encephalopathy, which are a major cause of treatment death [5, 18].

It is unclear whether decreasing the dose of IMRT during concomitant chemoradiotherapy improves survival in patients with locally recurrent NPC. In previous studies, concomitant chemoradiotherapy improved local tumor control and prolonged survival in patients with recurrent head and neck squamous cell carcinoma [19, 20]. To date, however, the effects of the addition of concomitant chemotherapy to radiotherapy on survival of patients with locally recurrent NPC have not been confirmed. In many studies, concomitant chemoradiotherapy was adopted due to its unique advantages in direct targeting of tumors and potential synergistic effects with radiotherapy. The role of concomitant chemoradiotherapy with cisplatin as the optimal treatment for newly diagnosed advanced NPC has been quite well established by a number of prospective studies and meta-analyses [8, 21–24]. Poon et al. [10] performed a retrospective analysis of 35 patients with locoregional recurrent NPC, of which 23 patients (66%) had rT3 or rT4 disease. In this cohort treated with concomitant chemoradiotherapy plus adjuvant chemotherapy, they observed a response rate of 58% (29% complete response and 29% partial response). The 5-year OS and progression-free survival (PFS) rates were 26% and 15%, respectively. Nakamura et al. [9] presented the outcomes of re-treatment of 36 patients with recurrent NPC using cisplatin-scheduled chemoradiotherapy. With a median follow-up of 40 months, the 3-year OS rate was 58.3%. Our data showed statistically significant survival benefits with the 3-year and 5-year OS rates of 68.7% and 41.8%, respectively, in the concomitant chemoradiotherapy group. Further subgroup analysis showed improved OS rates in patients with rT3–4 disease, stages III–IV disease, and a tumor volume >26 cm^3^. The potential explanations for these outcomes include the following: (1) the effects of concomitant chemoradiotherapy were not evident for NPC at early tumor stages and with small volumes; or (2) concomitant chemotherapy could yield long-term survival benefits for patients with advanced and bulky tumors by reducing the tumor volume and extent. Improved vascular distribution could improve drug transport, enhancing radiosensitivity and reducing nasopharyngeal mucosal necrosis.

The results of our study also suggest that concomitant chemoradiotherapy could improve the OS of patients with recurrence intervals of more than 30 months. This may be due to the increased tissue recovery time which could reduce the incidence of nasopharyngeal mucosal necrosis/massive bleeding and other fatal complications during repeated radiochemotherapy.

The total dose of re-irradiation was a key factor in determining the effects of repeated radiotherapy. Most studies have recommended a dose of at least 60 Gy since this is associated with improved control and/or survival during re-irradiation. Wang et al. [17] showed that, for early-stage disease, the 5-year OS rate was approximately 45% when more than 60 Gy was delivered to the tumor, whereas no patients survived for more 5 years when the dose was less than 60 Gy (*P* = 0.0001). Lee et al. [4] performed a retrospective analysis of 654 cases of recurrent NPC. In the study, the 5-year local control rate of early disease was 40%, 35%, and 14% when a biological effective dose (BED) of the second course was given at >70 Gy, 60–70 Gy, and <60 Gy, respectively. The hazard ratio for local failure decreased by 1.7% per BED (1 Gy) in the repeated course.

However, high re-irradiation doses are associated with severe late complications. Teo et al. [5] found that the severe complications caused by high-dose re-irradiation (radiation dose of more than 60 Gy) could outweigh the potential benefits for survival. They found a high 5-year incidence of serious late complications including trismus (69.9%) and temporal lobe necrosis (20.4%). Similarly, in a study of 86 patients treated with three-dimensional conformal radiotherapy, Zheng et al. [6] reported that the 5-year incidence of grade 3 and grade 4 late toxicities was up to 100% and 49%, respectively, when the mean dose to the tumor was 68 Gy in 34 fractions, and that toxicity was the main cause of death. Previous studies determined the occurrence of severe late toxicities after re-irradiation to be 6%–45% [5, 25, 26] and found that the rate of fatal late toxicities including nasopharyngeal mucosal necrosis/massive hemorrhage was 2.0%–40.6% [5, 12, 27]. Poon et al. [10] analyzed clinical data from 35 patients with locally recurrent NPC who were treated with cisplatin-based concurrent chemoradiotherapy plus adjuvant chemotherapy and found that the incidence of grades 3 and 4 late toxicity was 12% and 23% at 2 and 5 years, respectively, and included mainly temporal lobe necrosis (11% and 11%), cranial nerve palsy (6% and 6%), and endocrine abnormalities (6% and 18%).

In our study, the prescribed dose was 60 Gy for 27 fractions and the BED was approximately 66 Gy according to biological models. The incidence of grade 3 and grade 4 acute toxicity, mainly oral mucositis and dysphagia, was higher in concomitant chemotherapy group (17.6%) than in radiotherapy alone group (8.6%), although no patients halted radiotherapy. Serious late toxicities, such as nasopharyngeal mucosal necrosis, radiation encephalopathy, and cranial nerve palsies were similar between the two groups. The incidence of nasopharyngeal massive hemorrhage was significantly lower in the concomitant chemotherapy group than in the radiotherapy alone group (χ^2^ = 6.180, *P* = 0.013). Chemoradiotherapy might be able to shrink the tumor faster and accelerate the blood supply to the surrounding tissues, thus reducing the probability of nasopharyngeal mucosal necrosis. Therefore, IMRT with concomitant weekly cisplatin (i.e., concomitant chemoradiotherapy) is a treatment that could improve long-term outcomes in patients with locally recurrent NPC and should be studied as an alternative to IMRT alone.

In conclusion, concomitant chemoradiotherapy can improve OS of patients with locally recurrent NPC when compared with radiotherapy alone. Patients with advanced T category (rT3–4) and stage (III–IV) disease, recurrence interval >30 months, and tumor volume >26 cm^3^ could significantly benefit from concomitant chemoradiotherapy. Clinical trials with a large sample size are needed to fully assess survival rates and confirm the role of concomitant chemoradiotherapy in the treatment of locally recurrent NPC.
